# Regional Injection of CAR-T Cells for the Treatment of Refractory and Recurrent Diffuse Large B Cell Lymphoma: A Case Report

**DOI:** 10.3389/fcell.2020.00333

**Published:** 2020-05-08

**Authors:** Yan-Hui Wei, Yu-Zhuo He, Xiao-Yan Lin, Fu-Xian Ren, Hong-Bin Zhu, Ying Cheng, Zhen Nan, Zheng-Biao Liu, Jing-Ya Yu, Xue-Jun Guo

**Affiliations:** ^1^Department of Graduate School, Xinxiang Medical University, Henan, China; ^2^Puyang Oilfield General Hospital, Puyang, China

**Keywords:** chimeric antigen receptor T cells, diffuse large B cell lymphoma, refractory recurrence, regional injection, case report

## Abstract

**Background:**

Lymphoma is a common hematological malignancy with many subtypes and considerable heterogeneity. Traditional treatments include chemotherapy, radiotherapy, and surgery. Patients with relapsed, refractory or advanced stage lymphoma have a dismal prognosis. In recent years, chimeric antigen receptors (CARs) have been recognized as powerful tools that redirect antigen-specific T cells independent of human lymphocyte antigen (HLA) restriction and specifically kill tumor cells. Satisfactory results with CAR-based treatments have been achieved in relapsed/refractory B cell leukemia/lymphoma. Our center explored the strategy of subcutaneous injections combined with intravenous drip to overcome certain issues.

**Case presentation:**

A patient with stage IV refractory and relapsed diffuse large B cell lymphoma was treated with regional and intravenous CAR-T cells. During the observation period, the temperature of the skin at the abdominal wall mass was slightly elevated, and tolerable pain in the injection area was reported. Imaging showed regional liquefactive necrosis. After the sequential administration of ibrutinib and venetoclax, the abdominal wall mass significantly decreased in size.

**Conclusion:**

The regional injection of CAR-T cells might be safe and feasible for the treatment of regional lesions in patients with refractory and relapsed advanced lymphoma.

## Introduction

In June 2011, three cases of advanced chronic lymphocytic leukemia treated with chimeric antigen receptor-modified T (CAR-T) cells were reported with good curative effects (Porter et al., 2011). Since, CAR-T cell immunotherapy for relapsed and refractory lymphoma/leukaemia has developed rapidly worldwide (Maus et al., 2014; Kochenderfer et al., 2015; Zhu et al., 2016; Abramson et al., 2017; Locke et al., 2017; Neelapu et al., 2017; Schuster et al., 2017; Schmidts and Maus, 2018; Zhao et al., 2018). The conventional administration route both domestic and abroad is intravenous infusion, which is associated with various side effects, such as cytokine release syndrome, neurotoxicity, and even death (Lee et al., 2014; Neelapu et al., 2018), and this route is ineffective in some patients with local lesions. Our center administered CD19 CAR-T cells by injection into local lesions and intravenous infusion to a patient with recurrent local refractory lymphoma after several cycles of radiotherapy and chemotherapy. The results showed that local injection of the lesion was safe and well-tolerated, and the lesion became significantly smaller. These results might provide an important reference for the clinical application of CAR-T cells.

## Case Presentation

**FIGURE 1:**
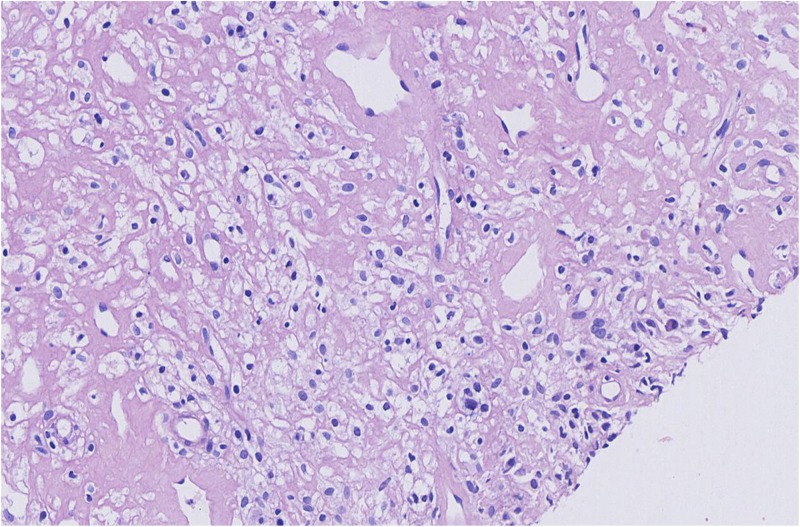
Microscopic findings for the biopsied specimen before the chemotherapy regimen was chosen. T cell/histiocyte-rich large B cell lymphoma.

A 57-year-old female patient with a weight of 70 kg presented at our hospital with a neck mass 5 years ago. She complained of neck discomfort in 2014 but was not prescribed medication. She presented again in May 2015 due to painless neck lymph node tumefaction without fever, for which she also did not receive medical treatment. By September 5, 2015, the bilateral lymph nodes in the neck had grown significantly, and more lymph nodes were affected. On September 8, 2015, a computed tomography (CT) scan showed tumefaction of bilateral cervical and supraclavicular lymph nodes, and the largest lymph node was 3.4 cm × 4.3 cm. Multiple enlarged lymph nodes were also found in the upper mediastinum, and the largest one was approximately 2.3 cm × 1.7 cm and occupied the thyroid. On September 11, 2015, PET/CT imaging showed bilateral cervical and supraclavicular lymph node involvement and multiple enlarged lymph nodes in the upper mediastinum (SUVmax: 9.05). Thyroid cancer with bilateral cervical lymph node metastasis or lymphoma was suspected, and cervical lymph node biopsy was performed. The pathological findings suggested T cell/histiocyte-rich large B cell lymphoma (Figure 1). The detailed immunohistochemical findings were as follows: CD20 (2+), PAX-5 (2+), ALK (−), BCL-6 (scattered weak+), CD10 (−), CD2 (−), CD21 (−), CD3 (−), CD30 (1+), CD4 (−), CD5 (±), CD56 (±), Ki67 (+60%), PD-1 (−), TIA1 (−), CD15 (−), LCA (3+), S-100 (−), CD68 (±), CD1a (−), and CD35 (−). The biopsy was negative for Epstein-Barr virus (EBV)-encoded RNA (EBER). The specific treatments are shown in Table 1.

**TABLE 1:** Treatment process and results.

Date	Treatment	Response
2015.10.16-2016.01.11	R-CHOP/4 cycles	PD
2016.01.16-2016.04.10	R-GDPI/4 cycles	SD
2016.04.15-2016.06.13	Etoposide + doxorubicin liposome + thalidomide/2 cycles	PD
2016.09.12-2016.10.07	Regional radiotherapy (20 Gy/20 times/25 days)	PR

The patient’s condition was stable during this period. On February 2017, a right abdominal wall mass was found, and a puncture biopsy was performed. Pathology revealed a T cell/histiocyte-rich large B cell lymphoma. On June 08, 2017, the abdominal wall mass was surgically resected because of ulceration. The postoperative pathological findings indicated lymphoma.		

2017.07.17-2017.12.25	MabThera + pemetrexed + dexamethasone/8 cycles	PR

2017.12.26-2018.06.10 The patient’s condition was stable, and clinical observation was performed at home. A mass on the right lower abdominal wall was again found on June 14, 2018.		

2018.06.28-2018.11.06	Pemetrexed + melphalan/6 cycles	2 cycles, PR4 cycles, PR6 cycles, PD
2018.11.17-2019.01.15	Cisplatin + isocyclophosphamide + etoposide + dexamethasone/2 cycles	PD

2019.01.14 Ultrasound examination: Soft subcutaneous swelling and uneven echo at the incision from the right lower abdomen to the right groin area was observed, and an irregular hypoechoic range of approximately 35 mm × 32 mm × 22 mm was visible at the lower edge of the incision. The boundary was unclear, and the local blood flow signal was increased. We considered the chemotherapy to be ineffective.		

2019.01.28-2019.02.26	Regional radiotherapy (40 Gy/20 times/29 days)	PR
2019.02.27-2019.04.14	Lenalidomide	PD
2019.04.15-2019.04.20	Lenalidomide + MabThera and local radiotherapy (10 Gy/5 times/5 days)	PD

**FIGURE 2:**
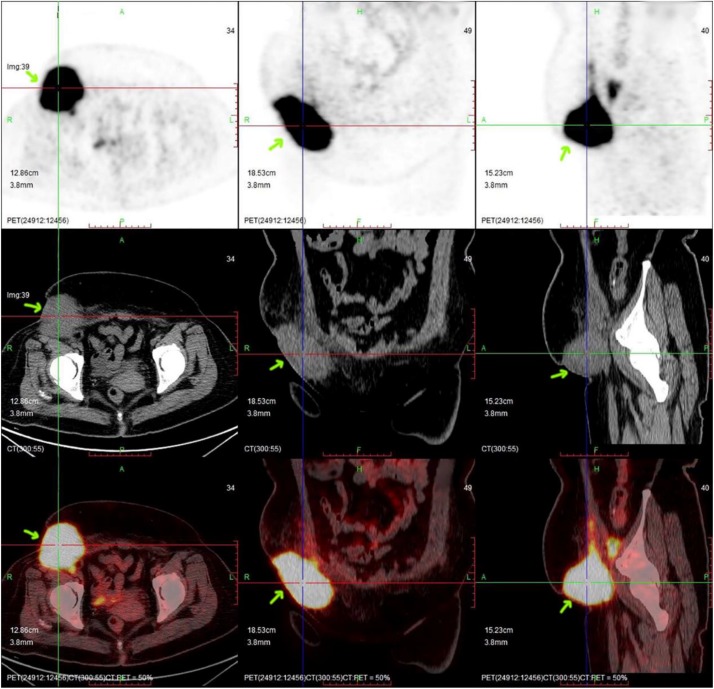
PET/CT imaging before CAR-T cell infusion. The position indicated by the arrow is the location of the lesion, which had a volume of ∼71 mm × 72 mm × 98 mm (arrow).

**FIGURE 3:**
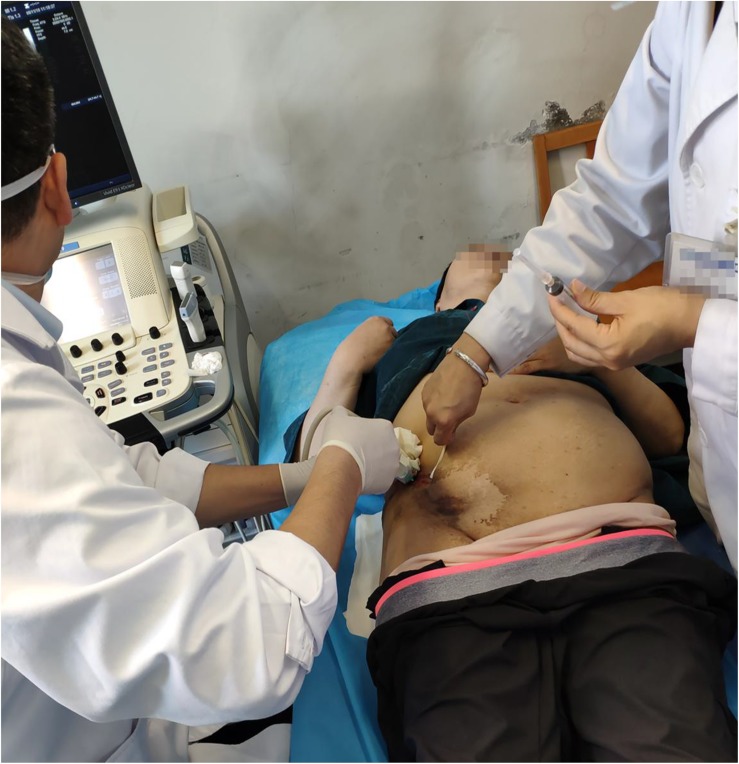
Picture of the local injection site.

**FIGURE 4:**
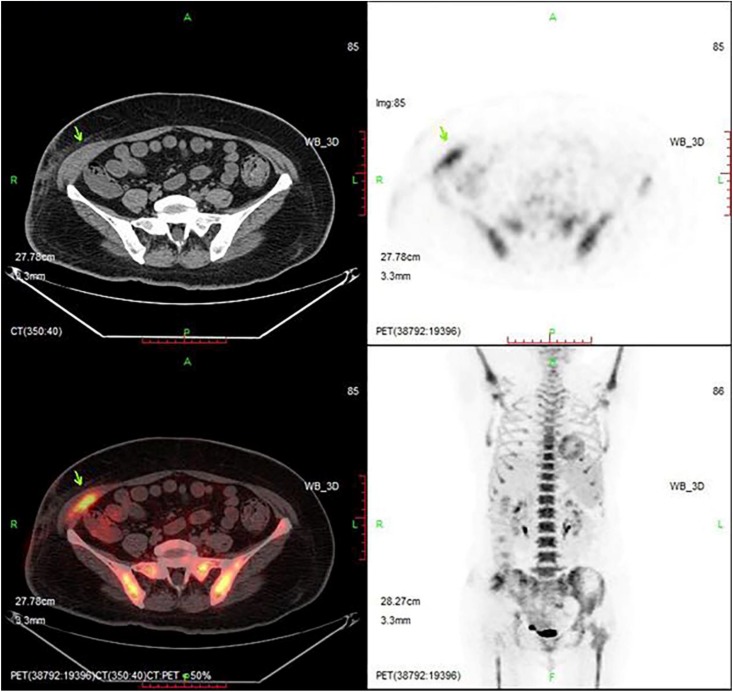
PET/CT imaging after CAR-T cell infusion. The lesion is significantly smaller than before. The position indicated by the arrow is the location of the lesion.

Re-examination by PET/CT showed multiple small and swollen lymph nodes in the right lateral iliac vessels and the right groin area that were partially fused into a clump, with a size of approximately 71 mm × 72 mm × 98 mm. The boundary with the adjacent abdominal oblique muscle and skin was unclear, the adjacent abdominal oblique muscle was thickened, the surrounding fat was turbid, and fluoro-deoxy glucose (FDG) metabolism was increased, with a SUVmax of 24.48. The right abdominal oblique muscle was invaded by lymphoma (Figure 2). Based on the above analysis, repeated systemic chemotherapy and local radiotherapy were applied, but the tumor still recurred in the local superficial area. This recurrence may be related to changes in the local anatomy of the patient after repeated treatment, and as a result, the drug could not reach an effective concentration locally. However, related chemotherapy drugs have no indication for local subcutaneous injection; therefore, CD19 CAR-T cell therapy was considered. On June 6, 2019, the baseline levels of cytokines (including IL-2, IL-4, IL-6, IL-10, TNF-α, and IFN-γ) were normal. An FC preconditioning regimen (fludarabine 30 mg d1-3 and cyclophosphamide 400 mg d1 + 600 mg d2) was administered on the same day, June 11, 2019, CD19 CAR-T cell treatment was started. Due to the local “barrier effect” of the lesion, we considered the possibility that the applied cells may not be able to affect the local area after intravenous infusion. Therefore, an approach of intravenous infusion combined with local injection was undertaken. Our center uses second-generation CD19 CAR-T cells generated by genetic engineering technology, in which the CAR domain that specifically recognizes tumor cells is transferred to activated T cells by lentivirus. The extracellular antigen-binding region is a single-chain variable region of a CD19-specific mouse antigen. With regard to the selection of intracellular costimulatory domains, related studies indicate that 4-1BB has better efficacy than CD28 (Zhi et al., 2018); therefore, our center utilizes the 4-1BB costimulatory domain. After the CAR-T cell therapy is constructed, it undergoes *in vitro* amplification and then is infused into patients. At present, the number of cells infused intravenously for this treatment at home and abroad is 1–10 × 10^6^/kg (Kochenderfer et al., 2015; Neelapu et al., 2017; Schuster et al., 2017). The total number of cells administered to this patient was 5.78 × 10^8^, and the following results were recorded: cell viability: 98.51% (trypan blue staining); cell marker detection: CD3 + CD4 + + CD3 + CD8 + > 95%; CAR19-positive rate: 63.65%; cell death activity: 48 h, F: T = 1:1, Raji: CD19 ratio < 20%. The cells (5.48 × 10^8^) were suspended in 100 ml of physiological saline for intravenous infusion, which proceeded normally, and the remaining 3.0 × 10^7^ cells were suspended in 10 ml of physiological saline for local treatment. According to the requirements for conventional surgery, under ultrasound guidance, we first selected an injection point in each of the four directions around the right lower abdominal wall mass. We injected 2 ml along each edge of the mass and then injected 2 ml in the center of the mass (Figure 3). The patient experienced no obvious discomfort except local swelling and pain. After 7 days of evaluation, the patient had no discomfort. The patient’s body temperature was 36.5°, the local skin temperature was 37.2°, and a scab appeared on the original skin mass with little exudation and was disinfected and covered with sterile dressings. The levels of the cytokines IL-6 and IL-10 in the blood appeared to slightly increase, 10.49 and 8.47 pg/ml, respectively, but the other factors were normal. At the evaluation on day 14, the patient complained of nocturnal pain in the mass, had a body temperature of 36.6° and a local skin temperature of 38.0°, and the levels of cytokines IL-6 and IL-10 had increased again (24.92 and 8.66 pg/ml), but the other factors were normal. At 16 days after the injection, ultrasonography detected a blurred mass of fused flakes (53 mm × 15 mm) with a slightly enhanced heterogeneous internal echo and abundant internal and surrounding blood flow in the right lumbar region and subcutaneous area of the right inferior abdomen. A cytological examination was suggested, but the patient requested to be discharged. Her out-patient treatment regimen was ibrutinib 400 mg/d + venetoclax 200 mg/d, and she reported a stable general condition. PET/CT imaging in August 2019 showed that the muscular tissue in the right antero-inferior abdominal wall had become thicker, with a slightly low-density shadow, unclear boundary and increased radioactivity uptake. The maximal cross-sectional area of the enhanced uptake shadow was 34 mm × 11 mm (SUVmax: 9.5), indicating increased tissue metabolism and a suspected malignant lesion. A regional flocculent, flaky, radiopaque shadow was observed in the subcutaneous areas of the right anteroinferior abdominal wall and right inguinal region (CT value: 27 Hu) with unclear boundaries. The radioactivity uptake was slightly increased (maximum SUV: 2.0), suggesting a higher level of metabolism. There was a soft tissue nodule beside the right lateral iliac artery with a maximum cross-sectional area of 20 mm × 8 mm (CT value: 54 Hu), slightly enhanced radioactivity uptake (SUVmax: 6.3), and increased metabolism, suggesting malignant lymph node lesions (Figure 4).

## Discussion and Conclusion

T lymphocytes play a key role in the cell-mediated immune response, and the body mainly relies on cytotoxic T lymphocytes (CTLs) to kill tumor cells. However, CTL function depends on antigen processing and presentation by MHC molecules (Reyburn et al., 1997), and these events are the main processes of tumor cell escape from immunosurveillance. CAR-T cells bypass the antigen presentation stage and MHC restriction and specifically kill tumor cells. There have been remarkable achievements in the treatment of relapsed/refractory B cell leukemia/lymphoma (Maus et al., 2014; Abramson et al., 2017; Kochenderfer et al., 2017; Locke et al., 2017). For our patient, the tumor recurred repeatedly under the local skin. Several imaging examinations indicated only regional invasion. The response to conventional radiotherapy and chemotherapy was poor, considering that the local anatomy of the patient may change after repeated treatment. The conventional methods of drug administration cannot achieve the effective concentration required for local treatment. Therefore, based on the treatment effect of the patient, it may be difficult to achieve the therapeutic effect only by the method of intravenous injection, so intravenous infusion combined with regional injection is used for treatment. During this process, the patient experiences local pain and an increase in skin temperature, which can be tolerated. Because of poor patient compliance, regional efficacy and cytological evaluations were not conducted in a timely manner. Relevant studies both at home and abroad suggest that after the completion of cell therapy, ibrutinib and venetoclax should be sequentially orally administered to the patient as part of the overall treatment regimen (Fraietta et al., 2016). Sixty days after the CAR-T cell injection, imaging examinations revealed that the mass in the original injection area had become significantly smaller. The patient developed local swelling, pain and fever during cell therapy, and the levels of the cytokines IL-6 and IL-10 increased to varying degrees. In addition, the local mass was clearly smaller, suggesting that the combined CD19 CAR-T cell strategy demonstrated a good response to the mass, and the side effects can be tolerated. However, we cannot completely exclude the synergistic effect of ibrutinib and venetoclax. Ibrutinib has been reported to have a synergistic effect with CAR-T cells (Fraietta et al., 2016; Long et al., 2017). Further analysis suggested that the regional injection and intravenous infusion of CAR-T cells for the treatment of refractory and relapsed advanced lymphoma did not cause serious local or systemic adverse reactions, indicating that local treatment is safer. In terms of efficacy, the results of this research are preliminary. Because of the use of ibrutinib, further research is needed to ascertain the specific efficacy of the CAR-T cells, and the specific injection dose, injection techniques, injection timin, g and combined medications will be the next focuses of our research. This treatment method provides a new option for the clinical application of cellular immunotherapy as well as a reference for the treatment of other solid tumors.

## Data Availability Statement

All datasets generated for this study are included in the article/supplementary material.

## Ethics Statement

This study was approved by the Ethics Committee of Xinxiang Medical College affiliated with Puyang Oilfield General Hospital (review number: 2019-04-0006-E01) and was performed with the consent of the patient and family members.

## Author Contributions

Y-HW participated in the entire process of research, data collection, operation log recording, and wrote the first draft of the manuscript. Y-ZH analyzed and evaluated the treatment and curative effects. X-YL supervised the experimental process and standardized the technical operation. X-JG guided the entire process in terms of theory and practice and revised the manuscript. All other authors revised the manuscript.

## Conflict of Interest

The authors declare that the research was conducted in the absence of any commercial or financial relationships that could be construed as a potential conflict of interest.

## Abbreviations

FDG: fluoro-deoxy-glucose
PD: progressive disease
PR: partial remission
SD: stable disease.

## References

[B1] Abramson J. S. Mcgree B. Noyes S. Plummer S. Wong C. Chen Y.-B. (2017). Anti-CD19 car T cells in CNS diffuse large-B-cell lymphoma. *N. Engl. J. Med.* 377 783–784. 10.1056/NEJMc1704610 28834486

[B2] Fraietta J. A. Beckwith K. A. Patel P. R. Ruella M. Zheng Z. Barrett D. M. (2016). Ibrutinib enhances chimeric antigen receptor T-cell engraftment and efficacy in leukemia. *Blood* 127 1117–1127. 10.1182/blood-2015-11-679134 26813675 PMC4778162

[B3] Kochenderfer J. N. Dudley M. E. Kassim S. H. Somerville R. P. T. Carpenter R. O. Stetler-Stevenson M. (2015). Chemotherapy-refractory diffuse large B-cell lymphoma and indolent B-cell malignancies can be effectively treated with autologous T cells expressing an anti-CD19 chimeric antigen receptor. *J. Clin. Oncol.* 33 540–549. 10.1200/jco.2014.56.2025 25154820 PMC4322257

[B4] Kochenderfer J. N. Somerville R. P. T. Lu T. Yang J. C. Sherry R. M. Feldman S. A. (2017). Long-duration complete remissions of diffuse large B-cell lymphoma after anti-CD19 chimeric antigen receptor therapy. *Mol. Ther.* 25 2245–2253. 10.1016/j.ymthe.2017.07.004 28803861 PMC5628864

[B5] Lee D. W. Gardner R. Porter D. L. Louis C. U. Ahmed N. Jensen M. (2014). Current concepts in the diagnosis and management of cytokine release syndrome. *Blood* 124 188–195. 10.1182/blood-2014-05-552729 24876563 PMC4093680

[B6] Locke F. L. Neelapu S. S. Bartlett N. L. Siddiqi T. Chavez J. C. Hosing C. M. (2017). Phase 1 results of ZUMA-1: a multicenter study of KTE-C19 anti-CD19 CAR T cell therapy in refractory aggressive lymphoma. *Mol. Ther.* 25 285–295. 10.1016/j.ymthe.2016.10.02 28129122 PMC5363293

[B7] Long M. Beckwith K. Do P. Mundy B. L. Gordon A. Lehman A. M. (2017). Ibrutinib treatment improves T cell number and function in CLL patients. *J. Clin. Invest.* 127 3052–3064. 10.1172/JCI89756 28714866 PMC5531425

[B8] Maus M. V. Grupp S. A. Porter D. L. June C. H. (2014). Antibody-modified T cells: CARs take the front seat for hematologic malignancies. *Blood* 123 2625–2635. 10.1182/blood-2013-11-492231 24578504 PMC3999751

[B9] Neelapu S. S. Locke F. L. Bartlett N. L. Lekakis L. J. Miklos D. B. Jacobson C. A. (2017). Axicabtagene ciloleucel CAR T-cell therapy in refractory large B-cell lymphoma. *N. Engl. J. Med.* 377 2531–2544. 10.1056/NEJMoa1707447 29226797 PMC5882485

[B10] Neelapu S. S. Tummala S. Kebriaei P. Wierda W. Gutierrez C. Locke F. L. (2018). Chimeric antigen receptor T-cell therapy – assessment and management of toxicities. *Nat. Rev. Clin. Oncol.* 15 47–62. 10.1038/nrclinonc.2017.148 28925994 PMC6733403

[B11] Porter D. L. Levine B. L. Michael K. Bagg A. June C. H. (2011). Chimeric antigen receptor-modified T cells in chronic lymphoid leukemia. *N. Engl. J. Med.* 365 725–733. 10.1056/nejmoa1103849 21830940 PMC3387277

[B12] Reyburn H. T. Mandelboim O. Valés-Gómez M. Davis D. M. Pazmany L. Strominger J. L. (1997). The class I MHC homologue of human cytomegalovirus inhibits attack by natural killer cells. *Nature* 386 514–517. 10.1038/386514a0 9087413

[B13] Schmidts A. Maus M. V. (2018). Making CAR T cells a solid option for solid tumors. *Front. Immunol.* 9:2593. 10.3389/fimmu.2018.02593 30467505 PMC6235951

[B14] Schuster S. J. Svoboda J. Chong E. A. Nasta S. D. Mato A. R. Anak O. (2017). Chimeric antigen receptor T cells in refractory B-Cell lymphomas. *N. Engl. J. Med.* 377 2545–2554. 10.1056/NEJMoa1708566 29226764 PMC5788566

[B15] Zhao Z. Chen Y. Francisco N. M. Zhang Y. Wu M. (2018). The application of CAR-T cell therapy in hematological malignancies: advantages and challenges. *Acta Pharm. Sin. B* 8 539–551. 10.1016/j.apsb.2018.03.001 30109179 PMC6090008

[B16] Zhi C. Runhong W. Qiuling M. Lin S. Feng H. Zixiao S. (2018). In Vivo Expansion and antitumor activity of coinfused CD28- and 4-1BB-engineered CAR-T cells in patients with B cell leukemia. *J. Mol. Ther.* 26 976–985. 10.1016/j.ymthe.2018.01.022 29503204 PMC6079368

[B17] Zhu Y. Tan Y. Ou R. Zhong Q. Zheng L. Du Y. (2016). Anti-CD19 chimeric antigen receptor-modified T cells for B-cell malignancies: a systematic review of efficacy and safety in clinical trials. *Eur. J. Haematol.* 96 389–396. 10.1111/ejh.12602 26115358

